# Burden of stroke attributable to selected lifestyle risk factors in rural South Africa

**DOI:** 10.1186/s12889-016-2805-7

**Published:** 2016-02-12

**Authors:** Mandy Maredza, Melanie Y. Bertram, Xavier F. Gómez-Olivé, Stephen M. Tollman

**Affiliations:** MRC/Wits Rural Public Health and Health Transitions Research Unit (Agincourt), School of Public Health, Faculty of Health Sciences, University of the Witwatersrand, Education Campus, St Andrews Road, Parktown, Johannesburg South Africa; Centre for Global Health Research, Umeå University, Umeå, Sweden; INDEPTH Network, Accra, Ghana

**Keywords:** Comparative risk assessment, Stroke, Rural, Body-mass index, Systolic blood pressure, South Africa

## Abstract

**Background:**

Rural South Africa (SA) is undergoing a rapid health transition characterized by increases in non-communicable diseases; stroke in particular. Knowledge of the relative contribution of modifiable risk factors on disease occurrence is needed for public health prevention efforts and community-oriented health promotion. Our aim was to estimate the burden of stroke in rural SA that is attributable to high blood pressure, excess weight and high blood glucose using World Health Organization’s comparative risk assessment (CRA) framework.

**Methods:**

We estimated current exposure distributions of the risk factors in rural SA using 2010 data from the Agincourt health and demographic surveillance system (HDSS). Relative risks of stroke per unit of exposure were obtained from the Global Burden of Disease Study 2010. We used data from the Agincourt HDSS to estimate age-, sex-, and stroke specific deaths and disability adjusted life years (DALYs). We estimated the proportion of the years of life lost (YLL) and DALY loss attributable to the risk factors and incorporate uncertainty intervals into these estimates.

**Results:**

Overall, 38 % of the documented stroke burden was due to high blood pressure (12 % males; 26 % females). This translated to 520 YLL per year (95 % CI: 325-678) and 540 DALYs (CI: 343-717). Excess Body Mass Index (BMI) was calculated as responsible for 20 % of the stroke burden (3.5 % males; 16 % females). This translated to 260 YLLs (CI: 199-330) and 277 DALYs (CI: 211-350). Burden was disproportionately higher in young females when BMI was assessed.

**Conclusions:**

High blood pressure and excess weight, which both have effective interventions, are responsible for a significant proportion of the stroke burden in rural SA; the burden varies across age and sex sub-groups. The most effective way forward to reduce the stroke burden requires both population wide policies that have an impact across the age spectra and targeted (health promotion/disease prevention) interventions on women and young people.

**Electronic supplementary material:**

The online version of this article (doi:10.1186/s12889-016-2805-7) contains supplementary material, which is available to authorized users.

## Background

Stroke is the second most common cause of death in South Africa, after HIV/AIDS and the leading cause of disability [1]. Recent estimates suggest that at least 30,000 strokes occur yearly in rural South Africa [2]. The major risk factors for stroke are common to other non-communicable diseases (NCDs) and are modifiable with effective interventions. They include high blood pressure, tobacco use, high blood glucose, physical inactivity, and overweight and obesity [3]. In an environment where health system resources to manage stroke victims are severely limited, consistent and up-to-date information on the number of premature deaths potentially prevented by changing the profiles of modifiable risk factors in a population is needed [4]. Such information will enable effective targeting of efforts and community oriented health promotion.

Increasing the nation’s life expectancy through prevention of premature deaths from NCDs and related lifestyle risk factors is a key health agenda for the South African government. This is the driving principle behind the five-year strategic plan called “Strategic Plan for the Prevention and Control of Non-Communicable Diseases 2013-17”, released in 2013 [5]. In that plan, 3 major components are identified to achieve this goal, including: healthy lifestyle promotion, health systems strengthening, and monitoring cases and risk factors to evaluate where the most population health improvements can be achieved. This study speaks to the third component. It builds on our previous work that showed that stroke as a marker condition for cardiovascular disease has increased significantly over the years in rural South Africa [2]. In that study, stroke related mortality of 114 per 100,000 person-years was significantly higher than that reported for comparable settings of sub-Saharan Africa (SSA) as was the morbidity as measured using the disability adjusted life year (DALY) metric [6]. Few other studies corroborate these findings and indicate that in general, NCDs as well as risk factors have increased rural South Africa [7, 8].

In the present study, we assess the relative contribution of key risk factors on stroke burden in a rural setting of South Africa. The intent is to identify the most important risk factors for stroke deaths and morbidity at the population level. Whilst useful data exists through the global burden of disease study of 2010 [9], it is aggregated at a regional level. The extent of cross-country and inter-country variations in risk factor exposure profiles warrant sub—national analyses that utilize best available and up-to-date local data. In South Africa, the most comprehensive attempt to quantify the impact of modifiable risk factors on disease including stroke was through the national burden of disease study of 2000 [10]. However, the analysis was not stratified by rural and urban and the data used is now out-of-date.

We employed a comparative risk assessment (CRA) strategy developed by the World Health Organization to quantify contributions of the modifiable risk factors to stroke mortality and morbidity [9, 11]. This methodology is well established and has been applied in the global burden of disease (GBD) studies, the South African national burden of disease study of 2000 and few other national and sub-national level studies [9–16]. CRA utilises local level data on risk exposures and cause-specific mortality, supplemented by data on risk factor –disease associations from large scale prospective studies to identify the proportion of deaths attributable to a risk factor. We derived the local data from the Agincourt Health and Demographic Surveillance System Site (HDSS), rural North-Eastern South Africa [17]. Data on risk-factor disease associations was derived from the GBD 2010 study [9]. To our knowledge, our results provide the first estimates of the stroke burden (mortality and morbidity) that could be averted by controlling the selected risk factors in rural areas of South Africa and provide crucial information that policy makers could use to determine the best course of action to effectively reduce the stroke burden in such settings.

## Methods

### Population and setting

This study was based on the Agincourt sub-district, a population of approximately 90,000 people, located in rural North-Eastern South Africa adjacent to Southern Mozambique [17]. The Agincourt sub-district is entirely covered by a health and socio-demographic surveillance system which has been in operation since 1992. Comprehensive data on deaths, migration and vital events, i.e. the underlying population denominator, has been collected through a yearly census for over 20 years. Detailed description of the study site is available elsewhere [17]. Agincourt sub-district is fairly typical of many of the rural communities of South Africa with high unemployment, high levels of labour migration, high HIV prevalence and low socio-economic status. The emerging evidence from the sub-district indicates that the population is undergoing a rapid health transition, characterised by an increase in non-communicable diseases (NCDs), stroke in particular [18]. Risk factors for NCDs are also on the rise with 40 % of women (30 % men), 15 years and older classified as hypertensive [8].

### Ethics approval

Ethical approval for the study was granted by the Committee for Research on Human Subjects (Medical) of the University of the Witwatersrand, Johannesburg, South Africa for both the MRC/Wits Rural Public Health and Health Transitions Research Unit’s (Agincourt) Health and Socio-Demographic Surveillance System and for specific add-on survey modules (Clearance certificate no. M131050).

### Selection of risk factors

Our key criteria for selecting risk factors followed those of the WHO CRA project [11]: (i) availability of reasonably complete data on risk factor exposures within the Agincourt sub-district, (ii) availability of data from high quality epidemiological studies that indicates the causal association of risk factor exposure to stroke mortality, and (iii) a potential for modification. We excluded risk factors such as total cholesterol for which the population mean was below the theoretical minimum for all age groups. Systolic blood pressure (SBP), body-mass index (BMI) and fasting plasma glucose were the risk factors chosen for final assessment.

### Estimating population attributable fractions

**Fig. 1:**
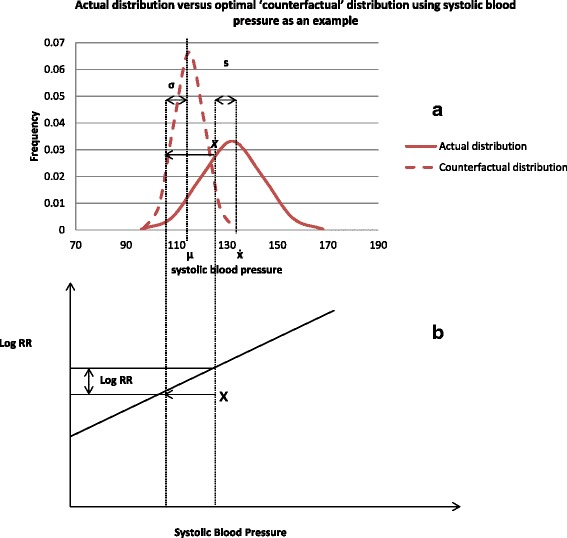
CRA methodology – comparison of population of interest with actual (factual) exposure distribution versus corresponding theoretical optimum distribution (the counterfactual) (**a**) and impacts on relative risk of disease occurrence (**b**)

We estimate the number of stroke deaths and disability adjusted life years (DALYs) that would have been averted in 2007-2011 in Agincourt sub-district if the current distribution of selected modifiable risk factors were shifted to an alternative optimal ‘counterfactual’ distribution as illustrated in Fig. 1 (using systolic blood pressure as an example). In that figure, the study population has an exposure distribution with a mean of x̄ and a standard deviation of *s*, compared to the ‘optimal’ (counterfactual) distribution with a mean of μ and standard deviation of σ. The non-zero standard deviation of the theoretical-minimum-risk distribution reflects the reality that there always is some inter-person variability within any given population, even after hypothetical reductions [11]. The lower part of Fig. 1 shows the distance (in SBP units) that random subject *x* travels on the exposure axis in moving to his or her corresponding position on the counterfactual distribution. This distance times the slope of log relative risk on SBP gives the proportional reduction in risk (i.e. the distance travelled on the ‘log RR’ axis), equivalent to the potential impact fraction (PIF) for this individual [19]. Summing the PIFs for all individuals gives us the population attributable fraction.

Mathematically the PAF for continuous risk factors is calculated as shown below:

Equation 1:

$\mathrm{Population}\ \mathrm{attributable}\ \mathrm{fraction}={\scriptscriptstyle \frac{{\displaystyle {\int}_l^h}RR\ (x)P(x)dx-{\displaystyle {\int}_l^h}RR(x){P}^{\hbox{'}}\ (x)dx}{{\displaystyle {\int}_x^h}RR\ (x)P(x)dx}}$ [9, 19] where: (i) *P*(*x*) = Current population distribution of risk factor exposure; (ii) *RR*(*x*) relative risk of mortality due to risk factor exposure; 0(iii) *P*′(*x*) is an alternative exposure distribution

Numerical integration was conducted in Excel, using customised spreadsheets as done in previous studies [19] and the spreadsheets are provided as (Additional file 1).

### Input data for PAFs

From Fig. 1 and equation 1, calculation of PAF requires four key input data:

#### Estimates of relative risk of stroke occurrence by risk factor

Age-specific relative risks for stroke occurrence by raised SBP, BMI and fasting glucose for South African population were extracted from the Global Burden of Disease study (Amy VanderZanden, personal communication). These estimates were derived by systematically reviewing and synthesising published and unpublished data. We assumed the same relative risks observed in South Africa would apply for the Agincourt population (Table 1).

**Table 1:** Age-specific relative risks of stroke occurrence due to raised systolic blood pressure (SBP) and body mass index (BMI) (GBD 2010 study estimates for South Africa, Amy VanderZanden - personal communication)

Risk factor	RR	25–29	30–34	35–39	40–44	45–49	50–54	55–59	60–64	65–69	70–74	75–79	80+
SBP	RR for 10 mmHg increase	2.17	2.07	1.97	1.88	1.8	1.71	1.64	1.56	1.49	1.42	1.36	1.26
	RR for 1 mmHg increase	1.08	1.08	1.07	1.07	1.06	1.06	1.05	1.05	1.04	1.04	1.03	1.02
	95 % CI	(2.03–2.31)	(1.95–2.20)	(1.87–2.09)	(1.79–1.99)	(1.71–1.89)	(1.64–1.79)	(1.57–1.71)	(1.50–1.62)	(1.44–1.54)	(1.38–1.46)	(1.32–1.39)	(1.24–1.29)
BMI	RR for 5 kg/m2 increase	1.88	1.81	1.74	1.68	1.61	1.55	1.49	1.44	1.38	1.33	1.28	1.21
	RR for 1 kg/m2 increase	1.13	1.13	1.12	1.11	1.10	1.09	1.08	1.08	1.07	1.06	1.05	1.04
	95 % CI	(1.69–2.08)	(1.64–1.99)	(1.59–1.91)	(1.54–1.82)	(1.49–1.74)	(1.45–1.67)	(1.40–1.60)	(1.36–1.53)	(1.31–1.46)	(1.27–1.40)	(1.23–1.33)	(1.17–1.25)

#### Prevalence estimates for the risk factors

We derived prevalence estimates from the HIV/NCD study “Ha Nakekela” - a cross-sectional study of 4,362 adults >15 years conducted in Agincourt HDSS in 2010 (Table 2) [8]. In that study, a team of experienced local fieldworkers collected the data on cardiometabolic risk factors using validated questionnaires and standardised measurement instruments. Physical measurements included height, weight and waist circumference using a flexible stadiometer (Seca, Hamburg, Denmark); and Analysis Scale Body Check (Seca, Hamburg, Denmark). Blood pressure was taken from an average of the last 2 measurements out of 3 readings taken 2 to 3 min apart using Boso blood pressure instrument (BOSCH + SOHN, Jungingen, Germany). Random blood glucose was measured with a Caresens POP blood glucose meter (i-Sens, Nowon-gu, Seoul Korea) [8].

**Table 2:** Prevalence estimates by sex and age group for systolic blood pressure (SBP) and body mass index (BMI) in Agincourt sub-district, South Africa, 2010 [8]

	Males			Females		
Age group	n	SBP	BMI	n	SBP	BMI
		Mean (SD)			Mean (SD)	
25–29	181	127.7 (9.7)	22.6 (3.6)	312	120.7 (10.1)	26.7 (5.6)
30–34	171	126.4 (10.6)	23.3 (4.3)	323	124 (11)	28.1 (6.3)
35–39	197	127.3 (11.8)	22.9 (4.4)	345	125.7 (12.1)	27.4 (6.1)
40–44	113	127.5 (13.7)	23.8 (4.8)	213	127.5 (14.1)	28.4 (6.4)
45–49	134	132.8 (13.9)	25.6 (7.9)	238	131.8 (13.3)	28.9 (7.6)
50–54	73	135.4 (10.5)	24.4 (4.9)	127	135.8 (13.6)	28.9 (7.2)
55–59	83	131.9 (14.1)	23.7 (5.1)	121	139.8 (14.7)	29.2 (6.6)
60–64	111	140.4 (13.6)	24.5 (5.2)	132	135.5 (12.2)	28.5 (6.8)
65–69	87	140.6 (15.9)	24.3 (4.5)	124	140.5 (14)	27.8 (5.7)
70–74	86	141.3 (13)	24.3 (5)	67	138.8 (11.4)	28.1 (6.8)
75–79	36	140.3 (13.5)	22.5 (4.3)	67	138.8 (12.4)	26.4 (5.3)
80+	52	137.9 (11.3)	23.7 (4.1)	66	143.7 (13.5)	25 (5.7)

Table 2 shows the prevalence of risk factors in Agincourt sub-district, South Africa, in 2010. The sample size (n) shown is for participants whose blood pressure was measured; this varied slightly more than the sample size for body-mass index

Because the number of individuals with blood glucose measures was small, we collapsed the age groups to form larger age bands (Table 3). Relative risk estimates are based on meta-analyses of prospective studies. This is the same data used by the global burden of disease study in 2010 [20].

**Table 3:** Mean plasma blood glucose levels and relative risk estimates by age and sex, Agincourt sub-district, 2010

Age group	Male	Females	
	Mean in mmol/L(SD) [8]		Relative risk of stroke occurrence(CI) [20]
35–44	4.32 (1.14)	4.26 (1.21)	1.19 (0.91–1.53)
45–54	4.43 (1.13)	4.64 (1.25)	1.16 (0.97–1.39)
55–64	4.26 (1.03)	5 (1.45)	1.14 (1.01–1.29)
65–74	5.26 (1.05)	5.04 (1.25)	1.14 (1.08–1.20)
75–84	4.70 (1.13)	4.75 (1.42)	1.1 (1.06–1.15)
85+	4.98 (2.48)	3.77 (1.77)	1.06 (0.98–1.16)

#### Estimates of theoretical minimum for the optimum ‘counterfactual’ distribution

For our base case, we have followed the GBD 2010 study in assigning exposure [9]. Based on previous meta-analyses of population-based studies, the mean (± standard deviation) of the optimal exposure distributions were chosen to be 23 (±1) kg/m^2^ for body mass index, 115 (±6) mmHg for systolic blood pressure and 4.9 (±0.9) mmol/l for fasting plasma glucose [9]. These are selected as the clinically meaningful exposures to minimize risk but these choices can be varied easily within the model. Worth noting is that the levels of blood pressure are consistent with levels reported in populations which have low cardiovascular disease, namely the Yanomamo Indians and rural populations in Kenya and Papua New Guinea [21].

#### Estimates of total stroke burden in study population

We obtained data on the number of stroke deaths by age and sex in 2007-2011 from the Agincourt HDSS. Cause of death in this sub-district is assigned through verbal autopsy, a process of interviewing a care–giver, relative or witness after a death, using a locally validated instrument [17]. The interview is conducted 1–11 months after a death and then reviewed independently by two medical doctors who assign probable underlying, immediate and contributory causes-of-death. When diagnoses differ and the physicians fail to reach a consensus, the verbal autopsy is reviewed by a third physician blind to the others’ assessments [17]. Further we calculated yearly incidence of stroke, years of life lost (YLL) due to premature mortality and disability adjusted life years (DALYs). The comprehensive details of this analysis are available elsewhere [2]. Further, based on estimates from a prospective community-based study conducted in rural Tanzania, we assumed 82.5 % of strokes were ischaemic [22] and focus on ischaemic strokes for this analysis. Because haemorrhagic stroke may well prevail in Agincourt sub-district, we vary this estimate in uncertainty analysis using data from Connor (2007) [23]. That study indicated that ischemic strokes comprise 71 % of total strokes in rural South Africa but this was a hospital-based study.

### Uncertainty analysis

We conducted statistical simulation to deal with uncertainty introduced by input parameters, namely relative risks estimates and prevalence of exposure data. We used @Risk 6.3 Professional for excel which allows multiple recalculations of a spread sheet, each time randomly choosing a value from distributions specified for input parameters (@RISK 6.3 Industrial) . We assumed that mean SBP, BMI and fasting glucose follow a normal distribution. Similarly relative risks followed a normal distribution, with the logarithm of the relative risks as the mean of the distribution. For each of the output variables (namely attributable burden due to BMI or high blood pressure), 95 % confidence intervals were calculated bounded by the 2.5th and 97.5th percentiles of the 1000 iterations generated.

## Results

Quantification of the contribution of risk factors on stroke occurrence in this analysis represents the effects of individual risk factors, holding other factors constant. The effects of multiple risk factors are not a simple addition of the individual effects and this should be borne in mind when interpreting the following results.

**Fig. 2:**
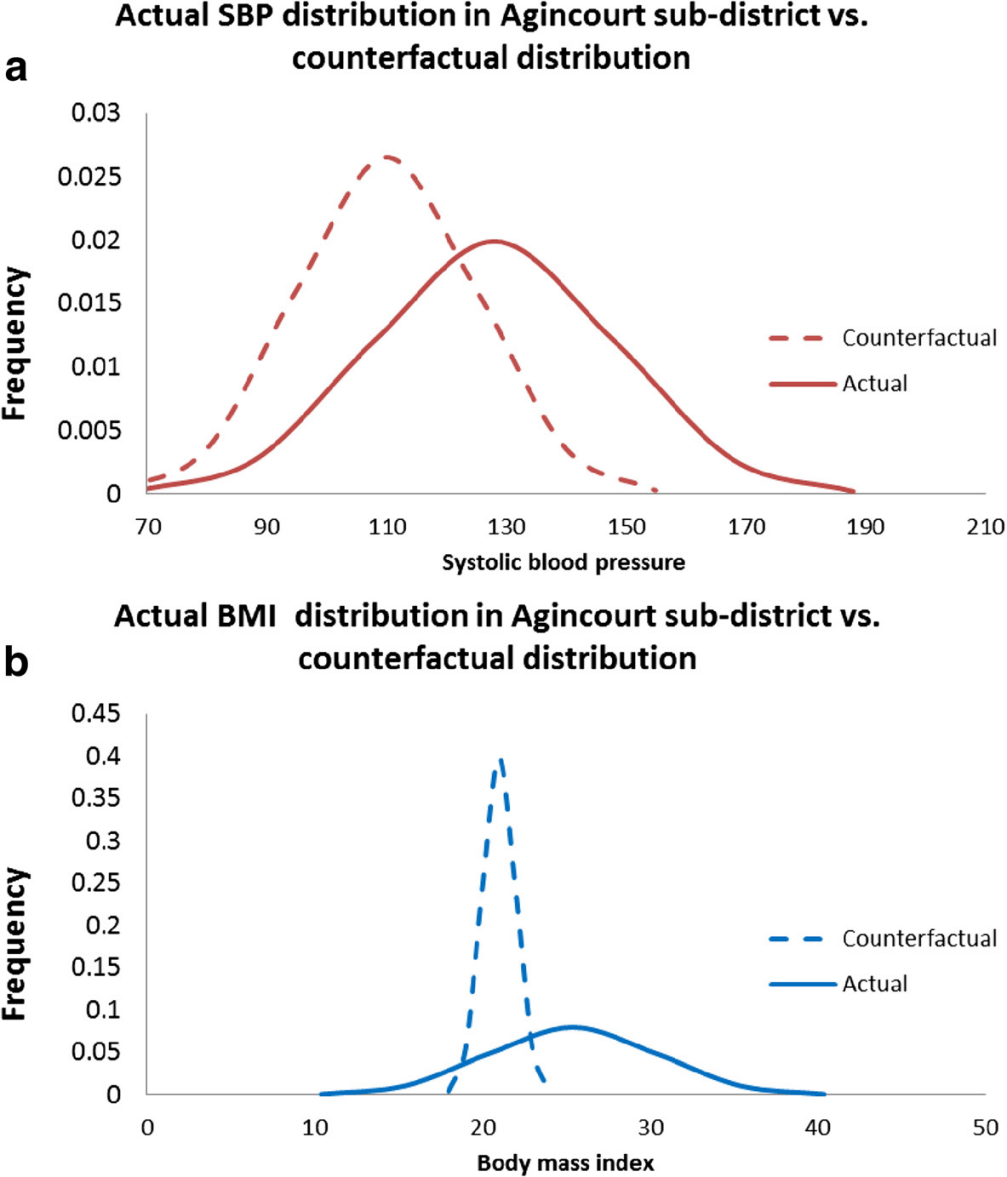
Graphical representation of comparative risk assessment methodology showing the actual distributions of systolic blood pressure (fig. 2 **a**) and body-mass index (fig. 2 **b**) in Agincourt sub-district, South Africa, 2010 compared with the targeted “counterfactual” distribution

The actual exposure distribution for SBP amongst individuals 25 years and older in Agincourt sub-district is shown in Fig. 2a. That distribution has a mean and standard deviation of 128 and 18 mmHg respectively. In accordance with the comparative risk methodology, ‘shifting’ this distribution to one that is clinically more beneficial (counterfactual) would have potentially avoided 40 % of the stroke burden (both mortality and morbidity) i.e. the population attributable fraction of SBP on stroke occurrence was 40 % in 2010 for both males and females. Similarly, ‘shifting’ the BMI distribution’ towards the counterfactual distribution (Fig. 2b) would have potentially prevented 22 % of the total mortality and morbidity burden experienced in Agincourt over the same time period.

**Fig. 3:**
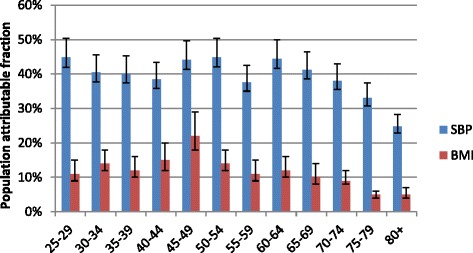
Distribution of PAFs for stroke due to SBP and BMI in adult males, Agincourt, South Africa, 2010

**Fig. 4:**
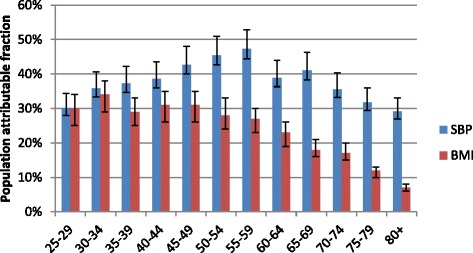
Distribution of PAFs for stroke due to SBP and BMI in adult females, Agincourt, South Africa, 2010

Population attributable fractions varied by age and sex within the sub-district (Figs. 3 and 4); this was more pronounced for SBP amongst females where PAFs increase steadily, peaking at 47 % at age 55-59, and then declining. For males, the distribution is less clear. However the proportion of stroke deaths/ DALYs due to high blood pressure is consistently high across all age groups, and average 35 %.

We used the same age-specific relative risk ratios for males and females for each risk factor. However, when comparing the burden attributable to BMI, there is a stark difference between males and females. Whereas the peak value for PAF in males was 22 % and occurred at ages 45-49 years; amongst females, PAFs for BMI were greater than 25 % for all ages less than 60 years. Worth noting is that in the younger age groups PAFs for BMI were more than double in females compared to males (11 % versus 30 %) in the age group 25-29 years. A similar pattern is observed up to age group 35-39 years. Overall, the proportion of deaths attributable to excess weight is highest in theage band 25-49 years amongst females compared to older age groups. However, because few deaths were classified as stroke-related in that age group in Agincourt in 2007 -11, the absolute number of stroke deaths attributable to BMI was small (i.e. 12 out of 38 deaths).

The PAFs for raised blood glucose were more erratic and comprised less than 5 % of the total burden of stroke. Challenges of measuring the fasting plasma blood glucose in the original study were documented and could partly explain this [8]. The results are thus not included here but shown in the Additional file 1; even so they should be interpreted with caution.

In terms of absolute numbers for the period 2007-11, high blood pressure was responsible for 520 YLL due to premature mortality (95 % CI: 325-678), of which 355 YLL (95 % CI: 221-467) occurred amongst females. Similarly, high blood pressure was responsible for a further 545 DALYs lost due to stroke (CI: 343-717). The burden attributable to high blood pressure was almost twice that of BMI (Table 4). Overall, there were more attributable YLLs and DALYs in females compared to males, meaning that by controlling high blood pressure and excessive weight, it is possible to markedly reduce the premature mortality from stroke amongst females.

**Table 4:** Stroke burden attributable to high blood pressure and body mass index (BMI) in males and females, Agincourt sub-district, South Africa, 2010

	Males		Females		Males		Females	
	SBP				BMI			
Age group	YLL	DALYs	YLL	DALYs	YLL	DALYs	YLL	DALYs
25–29	4.8	6.4	30.3	31.7	1.2	1.6	29.7	31.1
30–34	3.9	5.1	7.3	9.1	1.3	1.8	6.8	8.5
35–39	24.1	25.0	13.7	15.7	7.2	7.4	10.7	12.4
40–44	17.6	18.3	38.0	40.0	6.8	7.1	30.4	32.0
45–49	0.0	1.4	31.1	32.8	0.0	0.7	22.4	23.6
50–54	33.3	35.0	51.6	53.5	10.4	11.0	32.4	33.5
55–59	23.6	24.7	36.0	38.1	7.1	7.4	20.0	21.2
60–64	10.5	11.3	21.1	22.6	2.9	3.1	12.4	13.2
65–69	24.3	24.9	37.6	38.9	5.8	6.0	16.7	17.3
70–74	12.6	13.1	30.3	31.4	3.0	3.1	14.7	15.2
75–79	5.3	5.4	21.6	22.3	0.7	0.7	7.8	8.1
80+	5.6	5.7	36.9	38.1	1.1	1.1	9.1	9.4
25+	165.5	170.7	355.4	225.5	47.5	51.0	213.2	225.5

The table shows the number of years of life and DALY loss due to premature mortality that could have been prevented by shifting the population exposure distributions of systolic blood pressure (SBP) and body-mass index (BMI) from the current distribution observed in Agincourt to distributions that have been shown to be more clinically beneficial (optimal distribution). Those distributions will have means (SD) of 115 (6) mmHg and 23 (1) kg/m^2^ for SBP and BMI respectively

## Discussion

To the best of our knowledge, this is the first study to estimate the burden of stroke attributable to raised blood pressure, excess weight and raised blood glucose in rural South Africa, using an established methodology. Notwithstanding the inter-relationships between risk factors, the key strength of this approach is that it allows comparison of the relative contribution of modifiable risk factors on stroke burden thereby aiding public health prevention and promotion efforts. In addition, the analysis was based largely on local data derived from recent studies [8] with the exception of relative risk data which was based on meta -analyses of large prospective studies [20].

We have highlighted the very substantial burden of stroke incurred by increases in systolic blood pressure and adult BMIs with the greatest impact in terms of absolute numbers being the burden associated with raised blood pressure. This is consistent with findings of earlier studies which have ranked high blood pressure as the major risk factor for stroke deaths [24, 25]. The high prevalence of uncontrolled hypertension in South Africa could explain the contribution of raised blood pressure on stroke occurrence in the country [26]. These prevalence rates exceed 75 % and were the highest reported rates in the Study on Global Ageing and Adult Health (SAGE), which surveyed more than 35,000 people older than 50 years in South Africa, China, Ghana, India, Mexico and Russia [26].

Whilst the overall PAF values were lower than national estimates for South Africa produced in 2000 [25], age-specific ratios indicate that the proportion of deaths attributable to high blood pressure is much higher amongst the younger age groups in rural Agincourt. This is likely a ramification of higher prevalence of raised blood pressure within young adults. According to a landmark study by the Prospective Studies Collaboration, the effect of BMI and other vascular risk factors such as high blood pressure is far stronger at younger than at older ages [27, 28]. With regard to systolic blood pressure, a 20 mmHg lower SBP was associated with 64 % reduction in the risk of stroke death among individuals aged 40–49 years. In comparison, at ages 70–79 years the corresponding decrease in stroke was 50 % [16]. The mean systolic blood pressure amongst individuals aged 30-44 years was 122 mmHg in males and 116 mmHg in females in the South African National Burden of Disease (SA NBD) study [25]. In comparison, mean SBP was 127 mmHg and 125 mmHg amongst males and females respectively in Agincourt sub-district (author calculations based on data from the Agincourt HDSS).

Worth noting is that mean blood pressure was high, even when the proportion of the population classified as overweight was low amongst young males, suggesting that other factors contribute to increases in systolic blood pressure. These factors could include low birth weight, which is inversely associated with subsequent blood pressure, and is prevalent within the study population [29]. It has been estimated that a 1 kg higher birther weight is typically associated with a 2–4 mmHg drop in systolic blood pressure in 50-year-olds [30]. Nonetheless, we have no trend data on birth weight to support this explanation but these results warrant further exploration.

With respect to BMI, we show that burden of stroke attributable to this risk factor is concentrated amongst females and predominates in the younger age groups. Studies from elsewhere in the world corroborate this finding [26]. Similar to high blood pressure, the age-related differences in the PAFs can be attributed to the high prevalence of excess weight amongst young females, which increases the risk of cardiovascular disease [28].

Whilst the trend in age- and sex- specific PAFs in our study was similar to other studies [31–33], the absolute PAF values at younger ages were much higher in the current study even when prevalence rates of obesity were similar. A likely explanation is the difference in the “theoretical minimum risk” values applied across studies. In the current study we assumed that the optimal BMI distribution for a population that would confer minimum cardiovascular disease risk would have a mean of 23 kg/m^2^. In contrast, a multi-country analysis based on Asian populations used a higher mean of 24–25 kg/m^2^[31]. Had the latter study used a lower threshold as used in our study, the estimated PAFs would have been higher.

In light of our findings which indicate that considerable variation in the burden of stroke attributable to excess weight and raised blood pressure exists across age and sex groups, the anticipated benefits in terms of stroke prevention would differ across population subgroups. We therefore propose that intervening at two levels through population wide policy and targeted health promotion campaigns is the best way forward to effectively reduce the stroke risk in rural South Africa. Evidence suggests that a policy led approach to health promotion in relation to two broad food categories - salt and sugar would be cost-effective levers for a population wide health promotion approach due to the impact on SBP and BMI [34]. For example, for each 100 mmol reduction in sodium intake, an SBP reduction of 5–10 mmHg is expected, with variation according to age [35]. Further, the downstream impacts on stroke reduction are well documented, with a recent study based on South African data indicating that legislated sodium reduction could prevent some 2,900 fatal and 4,300 non-fatal strokes in South Africa and save the health system R300 million (US$35 million in 2012) [36]. Based on these and related estimates, the Minister of Health of South Africa, under section 15(1) of the foodstuffs, cosmetics, and disinfectants Act, 1972 (act 54 of 1972), gazetted the regulations relating to the reduction of sodium in certain foodstuffs [37]. The foodstuffs specifically mentioned include bread, butter spread, fat spread, processed meats, raw processed meat sausages and ready to eat savoury snacks. According to these regulations the first total sodium reductions should be effected by 30 June 2016; for bread that means total sodium content of bread should be 400 mg (per 100 g of bread) by that date. A further reduction of 20 mg should be achieved by June 2019. If implemented effectively, such a policy has the potential to reduce cardiovascular disease (CVD) burden in the country.

With regard to obesity, legislative options on sugar content which do not rely on individual behaviour change or a well-functioning health service offer an alternative population-wide approach. For example, a 20 % tax in sugar sweetened beverages was predicted to reduce obesity by 3.8 % (95 % CI: 0.6–7.1 %) in men and 2.4 % (95 % CI: 0.4–4.4 %) in women in South Africa [38]. However, the confidence intervals of that study were wide and suggest that the intervention would prevent less than 1 % of the obesity burden. More innovative research is needed in this area to assess interventions that could reduce cardiovascular diseases including stroke by targeting obesity.

In addition to population wide approaches, the implementation of interventions that target specific groups needs to be carefully developed. For example, “anti-obesity” campaigns specifically tailored towards young South African women may reap greater benefits than more generic campaigns aimed at the whole population. Similarly, pharmacological treatment for people whose risk of a cardiovascular event over the next 10 years is above 35 %, offers an alternative individual-based intervention that could reach the older age groups with fairly frequent encounters with the health system. Within the Agincourt sub-district other novel initiatives are being explored and one such example is the Nkateko (Hope) trial [39]. This cluster randomised trial, focused on integrated chronic care will evaluate the impact at community and clinic levels of using a clinic-based lay health worker, supervised by a nurse, in managing hypertension. There are other interventions that could effectively address high blood pressure and excess weight that we have not mentioned but the main message from this work is to use a combination of approaches so as to achieve maximal benefits - interventions tailored by population subgroup and population-wide policies.

## Conclusions

High blood pressure and excess weight, which both have effective interventions, are responsible for a significant proportion of the stroke burden in rural South Africa. Unless they are addressed through effective public health measures, the impact on the future stroke burden in rural South Africa will be substantial. When funds are limited, knowing which risk factor to target and whom to target in order to derive maximal benefit, is crucial for any informed decision making process. The current study provides this information within a rural South African context by applying an established comparative risk assessment methodology.

### Limitations

While this study provides the first estimates of burden of stroke attributable to key risk factors in rural South Africa, there are a few limitations. We elected not to report on the impact of HIV/AIDS on stroke occurrence in this HIV-prevalent setting due to limited data to support this analysis. The best available data that independently assessed HIV as a risk factor for stroke was based on a study from the Unites States [40]. Different genetic profiles and possibly, risk-factor disease associations between that population and the current study population challenged use of that data. We however show the “results” of that analysis in Additional file 1, and indicate that the uncertainty in the analysis was too high to allow any meaningful conclusions to be drawn. The joint effects of high blood pressure and BMI were not examined even though effects of high body-mass index are partly mediated through blood pressure. We chose not to assess the joint effects as the correlation between these risk factors is unknown and the grouped data could overestimate the burden from combined risk factors. We used BMI as a measure of overweight and obesity. However, other measures such as waist circumference and body fat are considered better predictors of CVD risk [19]. Available data was not sufficient to establish risk factor- disease relationships of these indicators and opportunity exists to examine use of such measures in future comparative risk assessment studies. In future, it will be important to assess the full spectrum of risk factors including alcohol, vegetable consumption and physical activity. Nonetheless, the results presented give us a snapshot into the impact of lifestyle risk factors on stroke burden in a rural South African setting undergoing rapid epidemiological transition.

## Additional file

Additional file 1:
**CRA methodology as implemented in Excel for Agincourt sub-district, South Africa.** (XLSX 643 kb)

## Abbreviations

BMI: Body-mass index
CRA: Comparative Risk Assessment
CVD: Cardiovascular disease
DALYs: Disability adjusted life years
GBD: Global Burden of Disease
HDSS: Health and Demographic Surveillance System Site
NCDs: Non-communicable diseases
PAF: Population attributable fraction
SBP: Systolic Blood Pressure
SD: Standard deviation
SSA: Sub-Saharan Africa
WHO: World Health Organization
